# Muscle synergy stability and human balance maintenance

**DOI:** 10.1186/1743-0003-11-129

**Published:** 2014-08-30

**Authors:** Tytus Wojtara, Fady Alnajjar, Shingo Shimoda, Hidenori Kimura

**Affiliations:** BTCC, RIKEN, Nagoya Japan

## Abstract

**Background:**

The signals that the central nervous system (CNS) produces and sends to the muscles to effect movement are not entirely understood. Muscle synergy theory suggests that the central nervous system produces a small number of signals that pass through a network that distributes combinations of these signals to the muscles. Though these synergies are rather stable over time, some variability is present.

**Methods:**

Here, we investigated the variability of muscle synergy and defined a synergy stability index (SSI) to quantify it. We measured the activity of muscles responsible for maintaining lateral balance in humans standing on a platform that was subjected to lateral disturbance from the platform. We then calculated muscle synergies attributed to postural reflex and automatic response by using non-negative matrix factorization (NMF). Finally, from the calculated muscle synergies, we obtained SSI.

**Results:**

We observed a positive proportional relation between balance performance and SSI. Participants who were adept at maintaining balance were found to have invariant muscle synergies, and non-adept participants showed variable muscle synergies.

**Conclusions:**

These results suggest that SSI can be used to quantitatively evaluate balance maintenance ability.

**Electronic supplementary material:**

The online version of this article (doi:10.1186/1743-0003-11-129) contains supplementary material, which is available to authorized users.

## Background

The term muscle synergy, also called motor synergy, neuromuscular synergy, or muscle mode, has been used in the literature extensively over the last decade. A muscle synergy is the activation of a group of muscles to contribute to a particular movement [1], thus reducing the dimensionality of muscle control. A single muscle can be part of multiple muscle synergies, and a single synergy can activate various muscles. Different ways of grouping muscles into synergies can be found in the literature [2]. Some researchers define synergies as muscle activation of a set of muscles contributing to a particular movement where each muscle contributes to only one synergy [3]. However, the existence of synergies is controversial, with studies providing evidence for and against the existence of muscle synergies [2]. Muscle synergies are calculated from electromyography (EMG) data, and have been described in frogs [4], cats [5], and humans [3, 6–8]. Studies have focused on voluntary movements such as walking [9], cycling [10], and upper-limb reaching [6]. Others have investigated reflexes in frogs by cutaneous mechanical stimulation [11] and in cats through displacement of a supporting surface [5], as well as automatic postural responses in humans, where upright stance synergies were identified for different directions [7].

Muscle synergies can be identified by factor analyses [12] such as non-negative matrix factorization (NMF) [13, 14] and principal component analysis (PCA) [15].

In this study, we calculated synergies from EMG data and used an NMF method based on the Lee–Seung algorithm [14], which is a basic and fast NMF algorithm with multiplicative updating rules.

We chose balance maintenance for our task to observe reflex and automatic responses of the central nervous system (CNS). We based our study on our previous work on human balance [16]. In a society where humans are living longer than ever before, deterioration of ability to maintain balance poses a serious problem for elderly persons [17]. The mechanism of balance maintenance must be clarified in order to find a solution to this growing problem.

Our hypothesis about the control architecture of the CNS is based on the bow-tie structure proposed in [18]. We regard this hypothesis as an assumption for the existence of synergies. Only a portion of the information about the environment outside the human body enters the sensory system. An even smaller portion of this information is relevant to a particular task, such as balance maintenance. The CNS processes this relatively small amount of information and sends signals to the muscles that, in turn, move the highly redundant musculo-skeletal structure. The bow-tie structure represents a model of reducing degrees of freedom, since it processes high-dimensional input and produces high-dimensional output by a rather low-dimensional processing unit. Dealing with low-dimensional data is very time-efficient and allows for processing multiple tasks at the same time.

We cannot measure the relevant synergy recruitment signals directly at source, that is, in the brain or other parts of the CNS. However, we can measure the outcome which is the muscle activation. From these data, we can estimate what signals the CNS must have sent. We call these signals synergies.

Since all muscles move the same body, and there are multiple muscles moving the same joint, most muscles work in groups. The muscle activation signals are related to each other and thus they can be represented by a smaller number of signals. From the EMG signals, we calculate these representative signals (synergy recruitment signals) and the weights (muscle synergies) of the network that distributes these signals to the muscles. The number of synergy recruitment signals and the number of synergies are therefore much smaller than the number of muscles used.

Here, we propose an index of synergy stability to quantify synergy variability. Our findings suggest that synergy stability can be a measure of task performance quality and can be quantified by using the metrics introduced hereafter.

## Methods

### Definition of muscle synergy

We assume that each EMG signal *M*_*j*_(*t*) from a muscle that produces a reaction to a disturbance of body posture can be described as

1 $$\begin{array}{c} M_{j}\left(t\right)=\overset{k}{\underset{i=1}{\sum }}w_{\text{ij}}C_{i}\left(t\right),\;\;\;j=1,\;\;\ldots \;\;,m \end{array}$$

where *m* denotes the number of measured muscles, *C*_*i*_(*t*) is the signal produced by the CNS to control the *i* th synergy, *w*_*ij*_ is a time-independent weight for muscle *j* in synergy *i*, and *k* is the number of synergies. We define *M*_*j*_(*t*) for the interval from 75 ms to 150 ms after disturbance onset. We chose this interval because reflex mechanisms and automatic responses are active within this time window.

### Lateral disturbance experiments

All experiments were approved by the RIKEN Ethics Committee.

**Figure 1:**
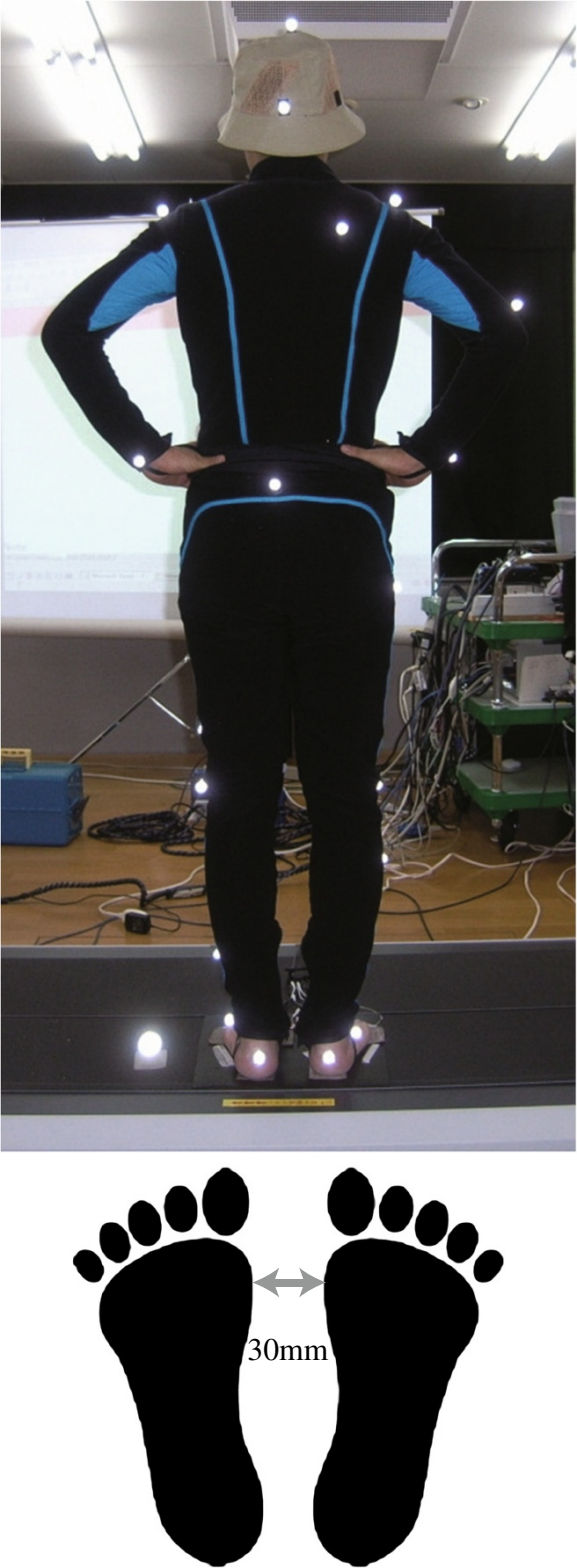
**Participant standing on the platform.**

Ten healthy persons (all men; age, 22–50 years; weight, 55–95 kg; height, 164–185 cm) participated in the experiment (Table 1). Participants stood on a platform with arms akimbo (on their hips) and feet parallel at about 30 mm apart, as shown in Figure 1. The arms akimbo position was used to cancel the effect of balancing using the arms. It is also closer to the natural hanging-down position than crossing arms on the chest, which made many participants feel uncomfortable. Crossed arms would also have obstructed optical markers placed on the chest for motion capture. Distance between the feet was set slightly shorter than shoulder width because participants did not show balance difficulties at greater distances. To effectively disturb the balance of a person with feet set at shoulder-width distance, the disturbance would have to be much stronger, which was technically infeasible and potentially dangerous.

**Table 1:** **Participant data**

Participant	Age	Height [cm]	Weight [kg]
sbj1	33	183	72
sbj2	40	172	68
sbj3	50	164	63
sbj4	28	185	95
sbj5	45	170	59
sbj6	35	170	60
sbj7	33	172	72
sbj8	30	178	73
sbj9	49	183	79
sbj10	22	165	55

During each trial the platform moved laterally as shown in Additional file 1. The timing and direction of movement were random and not known to the participants in advance. Movements in only the left direction were used for evaluation. During the movement of the platform to the left, the human body inclines to the right, putting body weight almost entirely on the right leg. The participants were instructed to stand still, keep their feet on the ground and avoid stepping and body movements other than lateral hip flexion/extension and ankle inversion/eversion. EMG signals of six muscles of the right side from 75 ms [19] to 125 ms after movement onset were recorded by using wireless active electrodes (BTS Bioengineering, Milan). The six muscles chosen for EMG recording were relatively easy-to-measure surface muscles in charge of lateral posture correction: muscles for ankle joint inversion, the flexor hallucius longus (FHL) and tibialis anterior (TA); for hip joint abduction, tensor faciae latae (TFL) and gluteus medius (GM); and for trunk lateral flexion, rectus abdominis (RA) and erector spinae (ES). Because RA produces a noisy signal when there is a fat layer over the abdominal muscles, only participants with relatively low fat were chosen.Additional file 1: Video showing balance maintenance experiments. (WMV 19 MB)

The following measures were implemented to create equal conditions for all participants and to keep them as motivated as possible.

We introduced a score system (see the subsection on assessment of inter-individual differences) to help participants maintain their concentration and motivation. The system provided a *platform score* (PS) that was displayed on a screen to give the participants feedback on their performance.

The participants stood on thin force sensors used to monitor lateral balance. The experiment was executed after confirming that the participants were balanced, that is, center of pressure was at the midpoint between the feet. This was done to prevent the participants from trying to predict the direction of the platform movement.

Muscle activation was monitored, and the experiment was carried out only when EMG activity was less than 30% of maximum, indicating a relaxed posture. This was done to prevent the participants from trying to predict the timing of the platform movement.

Two different disturbance strengths were used. The platform moved 160 mm within 0.66 s for the weak disturbance and 180 mm within 0.75 s for the strong disturbance. The parameters (displacement and duration) of the weak disturbance were tuned so as to produce a muscle response strong enough to obtain an acceptable signal to noise ratio but weak enough to not evoke a stepping response. The strong disturbance was used to determine differences in behavior between adept and non-adept participants for balancing. Adept participants were able to maintain their balance, while non-adept participants responded to the strong disturbance with a step or by lifting a leg.

### Calculation of muscle synergies from EMG data

EMG was sampled at 1 kHz, high-pass filtered with a 30 Hz cutoff, root-mean square rectified, and smoothed using moving average with sine function shaped window of length 10 samples.

The time window used for synergy extraction from EMG signals varies between studies. Many researchers investigating automatic postural responses base their methods on [20], where averages were taken from three time bins, two 75 ms long followed by one 350 ms long, starting 75ms after onset of a disturbance. Similarly in [7], the authors used three 75-ms time bins after disturbance onset. A finer time division was also applied for voluntary movements in [8].

Muscle activity signals *M*_*j*_(*t*) (Eq. 1) constituted the experimental data and were arranged to form an *m*×*n* matrix *M*, where *m* denotes the number of measured muscles and *n* the number of samples. EMG data *M* were then factorized by NMF to obtain the synergy matrix *W* and the synergy recruitment matrix *C* such that

2 $$M_{m\times n}=W_{m\times k}C_{k\times n}+E_{m\times n},$$

where *k* denotes the number of synergies and *E* the residuals. EMG data from each trial were used as input to the NMF algorithm. We selected the Lee–Seung algorithm [14] for our NMF, as other algorithms tended to be unstable. The initialization matrix of the NMF algorithm was set randomly, and the calculation was run 10 times for each data set. No significant changes in the results were observed.

Each of the 10 participants performed total of 12 left-direction and 12 right-direction trials in random order. Four left-direction trials per person were selected for further synergy calculation. Those were trials where the participants did not expect the disturbance and were relaxed. Specifically, they showed balanced posture, equal values for the foot sensors, and low EMG activity shortly before the onset of the disturbance. On the basis of observations and questioning of the participants, we decided to treat the first trials as a warm up and excluded them from evaluation. Data from one participant who reported fear and fatigue on the day of the experiment and performed many unnecessary recovery movements were discarded as outliers. Data from the remaining 9 participants were used for evaluation.

### Number of synergies

We calculated the variability accounted for (VAF) to determine to what extent the original EMG data could be reconstructed from the factorized data. The VAF is defined as

3 $$\text{VAF}=1-\frac{{∥E∥}_{F}^{2}}{{∥M∥}_{F}^{2}},$$

where ∥·∥_*F*_ denotes the Frobenius norm of a matrix. VAF calculations were carried out for each number of synergies 1 to *m*. When VAF was higher than the 90*%* threshold, then the number of synergies was deemed sufficient to regard the factorization results *W* and *C* as representative of the data set *M*[21, 22].

### Assessment of inter-individual differences

To assess inter-individual differences, we introduced two quantitative indexes of balance ability, *synergy stability index* (SSI), based on synergy calculations and *platform score* (PS), based on observations of human balance recovery.

#### Synergy stability index

To obtain an SSI for each participant, synergies were calculated for each weak disturbance trial, and their variability was evaluated by calculating the correlation between the synergies of that participant.

The rows of the matrix *W* are the synergy vectors, and we define *w*_*j*_(*j*=1,…,*p*) as the synergy vector of a participant for the *j* th trial for a particular synergy. If *w*_*j*_ are well aligned, or more precisely, if *w*_1_=*w*_2_=…=*w*_*p*_=*w* after normalization, then we can say that *w* is the synergy vector of this participant for the experiment. In reality, synergy vectors are not identical from trial to trial, and we evaluate the degree of alignment among synergy vectors of different trials, representing this by the Pearson’s correlation coefficient *r* (Eq. 5) for each of the synergy vector pairs. Results were averaged over all *p*(*p*−1)/2 pairs, and then the averaged pairwise correlations were further averaged with respect to synergies to yield SSI. More precisely, SSI is represented as

4 $$\text{SSI}=\frac{1}{k}\overset{k}{\underset{i=1}{\sum }}\left[\frac{2}{p\left(p-1\right)}\overset{p}{\underset{l\neq q}{\sum }}r\left(w_{l}^{\left(i\right)},w_{q}^{\left(i\right)}\right)\right],$$

where *p* is the number of trials, *k* is the number of synergies, and $w_{l}^{\left(i\right)}$ and $w_{q}^{\left(i\right)}$ are the *i* th normalized synergy vectors of the *l* th and *q* th trials, respectively. Synergy vectors were normalized such that the sum of all elements squared equaled 1. We sorted the resulting synergies to determine the highest SSI. The Pearson’s correlation coefficient *r* is defined as

5 $$r\left(x,y\right)=\frac{\sum_{j=1}^{m}\left(x_{j}-\bar{x}\right)\left(y_{j}-\bar{y}\right)}{mS_{x}S_{y}},$$

where *x* and *y* are two vectors to compare; $\bar{x}$ and $\bar{y}$ are mean values, while *S*_*x*_ and *S*_*y*_ are standard deviations.

#### Platform score

Balance ability was also assessed by observing participants behavior during balance recovery from the strong disturbance and quantitatively expressed by PS. All trials were used for calculation so as to evaluate the overall ability of a participant and not a single strategy as in SSI calculation.

The maximum score was achieved when participant stood on both feet and kept both hands in the akimbo position at all times during the disturbance. Scores were penalized for the following behaviours. Removing one hand from the akimbo position was penalized one point, and removing both hands from the akimbo position was penalized two points. Lifting one heel off the surface was penalized one point, completely lifting one foot off the surface was penalized two points, and stepping aside with one foot was penalized three points. Totally losing balance and stepping aside with both feet cost the participant all six points.

## Results and discussion

**Figure 2:**
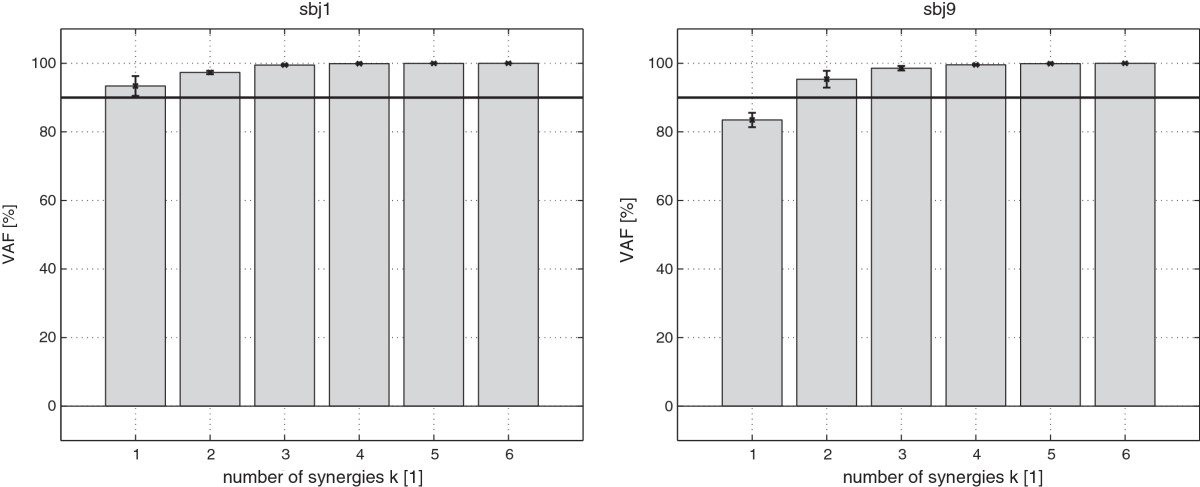
**VAF.** Two examples of VAF. SSI for number of synergies k = 1… 6 are shown. Participant sbj1 (left) has one synergy since his SSI for *k*≥1 is greater than the 90*%* threshold (horizontal line), and participant sbj9 (right) has two synergies since his SSI for *k*≥2 is greater than the threshold. Error bars denote standard deviation.

VAF calculation (Figure 2) revealed that the number of synergies *k* needed to reconstruct the original muscle activity data of all measured muscles was one or two, depending on the participant. Thus, we extracted an equal number of synergies (two) across participants to facilitate comparison. This low number of synergies can be explained by the fact that the human body in the lateral direction can be modeled with one degree of freedom for weak disturbances and two degrees of freedom for strong disturbances. For weak disturbances, the ankle joint is sufficient to maintain balance (ankle strategy); for strong disturbances, the ankle together with the hip joint play the main roles in balance maintenance (hip strategy) [23].

**Figure 3:**
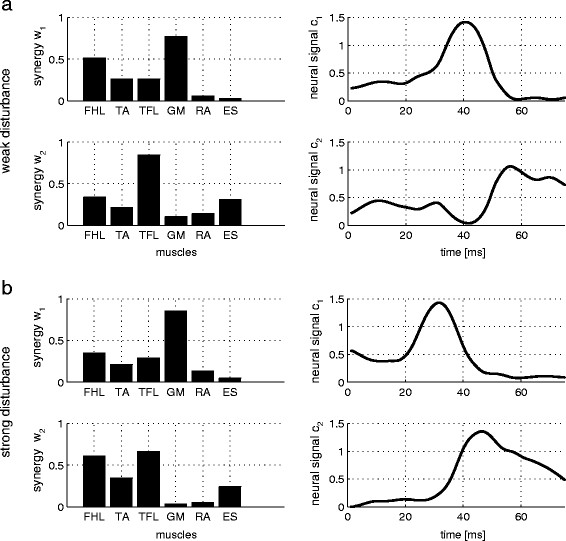
**Synergy.** Two sets of synergies *W* (bar graphs) and synergy recruitments *C* (time plots) for participant sbj6. **a**: weak disturbance; **b**: strong disturbance.

Figure 3a shows an example set (sbj6) of synergy *W* and synergy recruitment *C*. Each of the two synergy vectors *W* can be found in a separate bar graph on the left side. Each of the two synergy recruitment signals *C* is presented in a separate graph on the right side.

**Figure 4:**
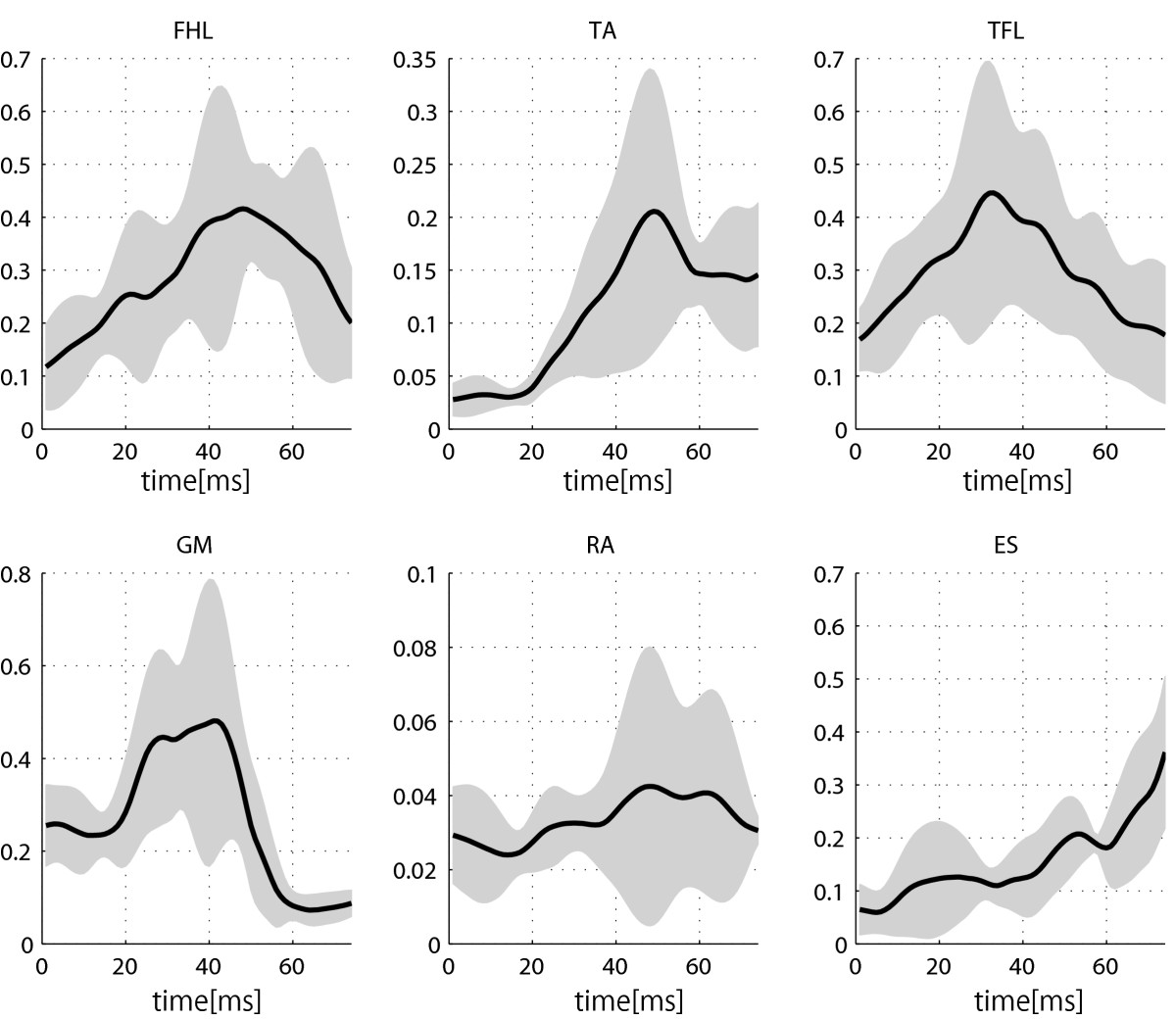
**EMG data of six muscles of participant sbj6.** Mean EMG activity (solid line) and standard deviation (gray area) for Flexor hallucius longus (FHL), tibialis anterior (TA), tensor faciae latae (TFL), gluteus medius (GM), rectus abdominis (RA), and erector spinae (ES) are shown.

**Figure 5:**
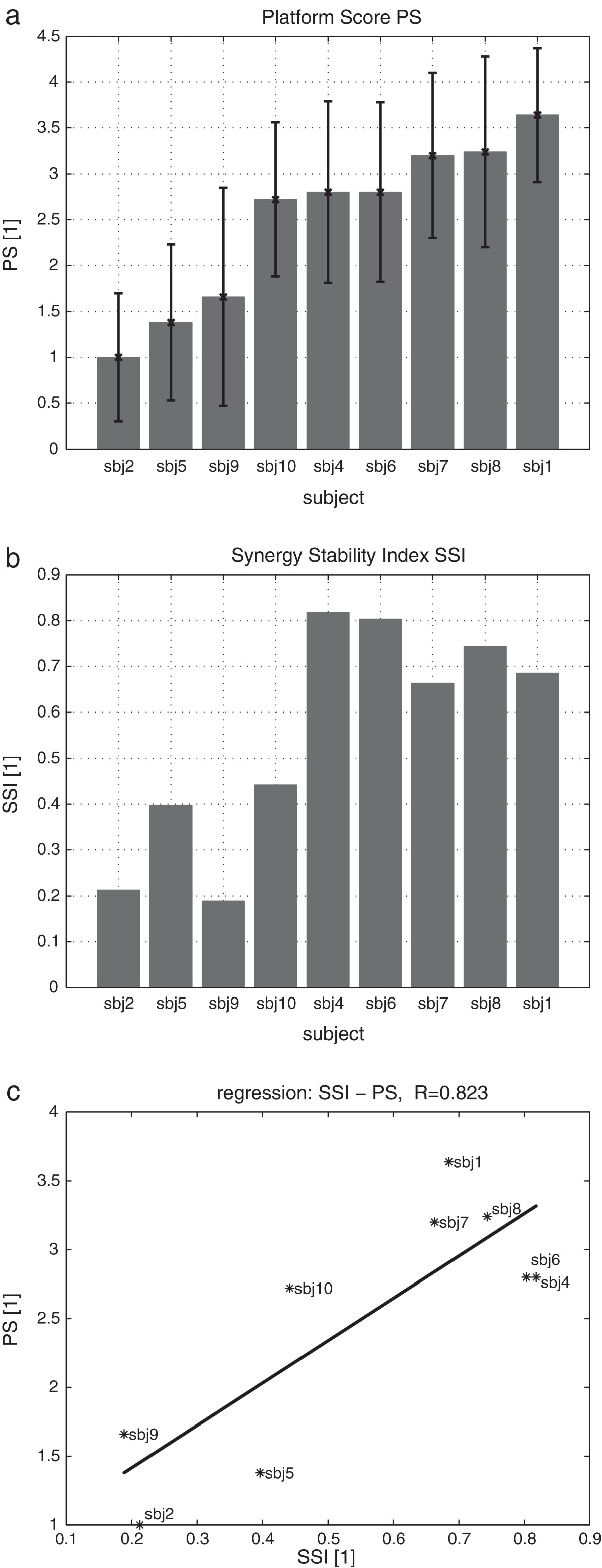
**PS and SSI. a**: PS of nine participants from the strong disturbance; **b**: SSI of nine participants from the weak disturbance; **c**: Relation between SSI and PS, with linear least squares regression (solid line).

**Figure 6:**
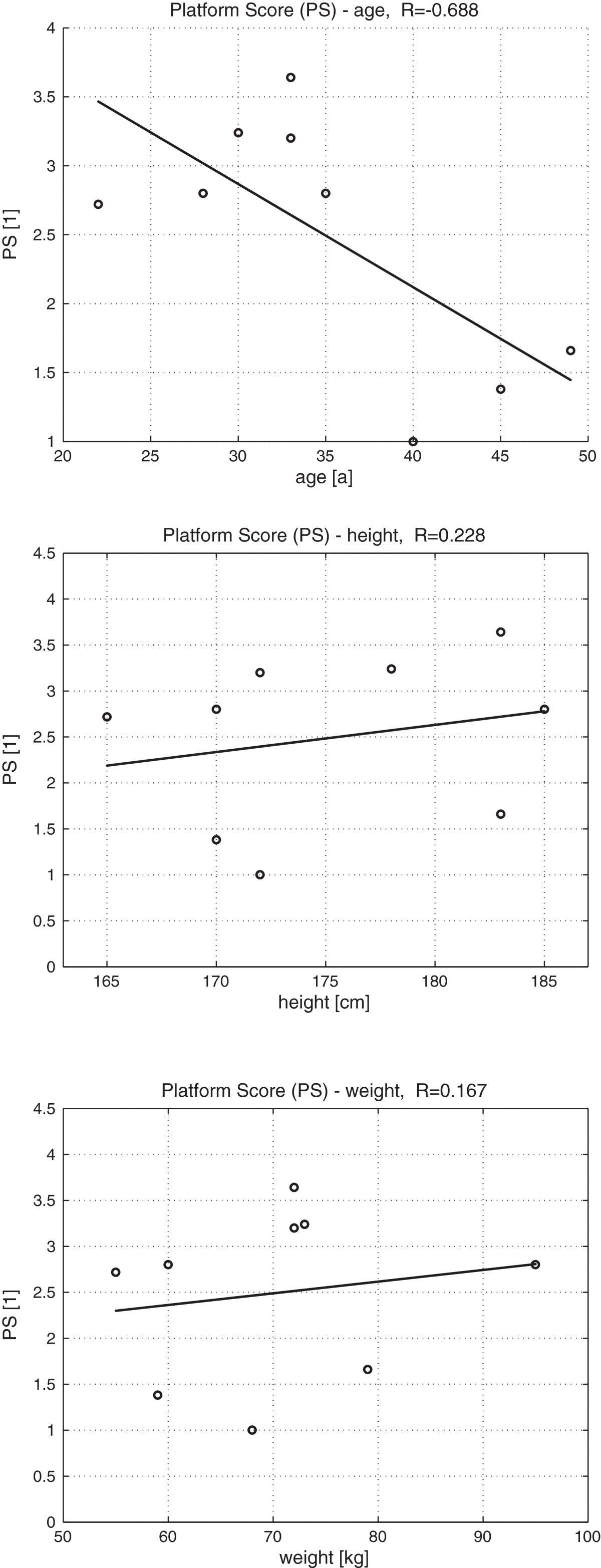
**PS as function of age, weight, and height.** PS as a function of age, weight, and height, with linear least squares regression (solid line).

Mean EMG values and the standard deviations of all six measured muscles (Figure 4) revealed that muscle activation can vary from trial to trial. The synergy similarity among trials is expressed by the SSI. Its values, for each participant, can be seen in Figure 5b. However, despite inter-trial differences among EMG signals, inter-trial synergies revealed to be similar as expressed by SSI values (Figure 5b). PS mean and standard deviation (Figure 5a) were calculated from approximately 12 platform movements for each participant and provided a quantification of balance performance. PS was not found to be dependent on height or weight and to be only slightly dependent on age (Figure 6).

Plotting PS as a function of SSI for each participant (Figure 5c) and applying linear least squares regression revealed a strong correlation (*R*=0.82) for the nine participants. This indicates that participants adept at maintaining balance (high PS) used the same synergy (high SSI), while non-adept participants used different synergies (low SSI) across trials. In other words, it can be said that persons who have high PS also have high SSI, and vice versa, as can be seen in Figure 5c and by comparing Figure 5a with Figure 5b. Therefore, participants who were able to minimize synergy variability were the most adept at maintaining balance.

PS was derived from strong disturbance trials to allow observation of differences in balance ability between participants. The strength of the disturbance was chosen such that some participants were not able to maintain their balance without waving their arms or stepping aside. SSI, in contrast, was derived from weak disturbance experiments. At this strength of movement, all participants were able to maintain balance without arm movements or stepping. One participant who was adept at balancing for both weak and strong disturbances showed similar synergies for the two disturbances (Figure 3a and b, left). Differences in muscle activity (Figure 3a and b, right) can be attributed to the synergy recruitment *C*.

The results suggest that the CNS is not able to produce a constant synergy in persons who are not adept at maintaining balance. Adept persons, on the other hand, will show less variations between disturbances. We assume that during balance training the CNS tries to adjust its synergy within a certain range to find the best configuration. In non-adept persons, this synergy is adjusted in a wider range than in persons who are adept at maintaining balance.

Our study was not without limitations. Our balance scoring system was not a clinically established index of balance. However, we were forced to use a self-designed scoring system because established indexes didn’t fit our needs. The Berg Balance Scale, for example, is a well established index that neglects dynamic balance and was designed for persons with balance disabilities while we dealt with healthy persons and needed an index to examine their dynamic balance. Furthermore, EMG data for the ES muscle tended to have noise due to sweat on the lower back as a result of physical exertion during the experiment.

## Conclusions

We applied a platform disruption method to investigate the neural mechanisms of involuntary motor control. Muscle synergies with distinct space-time separation were found, suggesting a possible method for reduction of muscle redundancy in motor control. We measured muscle activity of reflex and automatic posture control, then computed the associated muscle synergies. Analysis revealed that some participants showed relatively constant synergy across trials, while others showed different synergies. We demonstrated that participants with constant synergy were more adept at maintaining balance after lateral perturbation than those with different synergies among trials. This suggests the potential for the use of synergy constancy represented by SSI as a measure of ability to maintain balance or neural control quality.

## Consent

Written informed consent was obtained from the patient for the publication of this report and any accompanying images.

## Electronic supplementary material

### Authors’ original submitted files for images

Below are the links to the authors’ original submitted files for images.Authors’ original file for figure 1Authors’ original file for figure 2Authors’ original file for figure 3Authors’ original file for figure 4Authors’ original file for figure 5Authors’ original file for figure 6

## References

[1] Cordo PJ, Bell CC, Harnad S (1997). Motor Learning and Synaptic Plasticity in the cerebellum.

[2] Tresh MC, Jarc A (2009). The case for and against muscle synergies. Curr Opin Neurobiol.

[3] Shima K, Tsuji T (2010). Classification of combined motions in human joints through learning of individual motions based on muscle synergy theory. Proceedings of the 2010 IEEE/SICE International Symposium on System Integration (SII 2010).

[4] Kargo W, Ramakrishnan A, Hart C, Rome L, Giszter S (2009). Simple experimentally-based model using proprioceptive. J Neurophysiol.

[5] Ting LH, McKay JL (2007). Neuromechanics of muscle synergies for posture and movement. Curr Opin Neurobiol.

[6] d’Avella A, Portone A, Fernandez L, Lacquaniti F (2006). Control of fast-reaching movements by muscle synergy combinations. J Neurosci.

[7] Torres-Oviedo G, Ting LH (2007). Muscle synergies characterizing human postural responses. J Neurophysiol.

[8] Cheung V, Piron L, Agostini M, Silvoni S, Turolla A, Bizzi E (2009). Stability of muscle synergies for voluntary actions after cortical stroke in humans. PNAS.

[9] Ivanenko YP, Poppele RE, Lacquaniti F (2004). Five basic muscle activation patterns account for muscle activity during human locomotion. J Physiol.

[10] Wakeling JM, Horn T (2009). Neuromechanics of muscle synergies during cycling. J Neurophysiol.

[11] Tresh MC, Saltiel P, Bizzi E (1999). The construction of movement by the spinal cord. Nat Neurosci.

[12] Merkle LA, Layne CS, Bloomberg JJ, Zhang JJ (1998). Using factor analysis to identify neuromuscular synergies during treadmil walking. J Neurosci Methods.

[13] Cichocki A, Zdunek R, Phan AH, Amari S (2009). Nonnegative Matrix and Tensor Factorizations: Applications to Exploratory Multi-way Data Analysis and Blind Source Separation.

[14] Lee D, Seung S (2001). Algorithms for non-negative matrix factorization. Adv Neural Inf Process Syst.

[15] Tresch MC, Cheung VCK, d’Avella A (2006). Matrix factorization algorithms for the identification of muscle synergies: evaluation on simulated and experimental data sets. J Neurophysiol.

[16] Wojtara T, Sasaki M, Konosu H, Yamashita M, Shimoda S, Alnajjar F, Kimura H (2011). Artificial balancer - supporting device for postural reflex. Gait Posture.

[17] Vellas B, Toupet M, Rubenstein L, Albarede JL, Christen Y, Albarede J (1992). Falls, Balance and Gait Disorders in the Elderly.

[18] Csete M, Doyle J (2004). Bow ties, metabolism and disease. Trends Biotechnol.

[19] Clair JM, Okuma Y, Misiaszek JE, Collins DF (2009). Reflex pathways connect receptors in the human lower leg to the erector spinae muscles of the lower back. Exp Brain Res.

[20] Horak F, Diener H, Nashner L (1989). Influence of central set on human postural responses. J Neurophysiol.

[21] Frere J, Hug F (2012). Between-subject variability of muscle synergies during a complex motor skill. Front Comput Neurosci.

[22] Torres-Oviedo G, Macpherson JM, Ting LH (2006). Muscle synergy organization is robust across a variety of postural perturbations. J Neurophysiol.

[23] Runge C, Shupert C, Horak F, Zajac F (1999). Ankle and hip postural strategies defined by joint torques. Gait Posture.

