# Supplementary figures and images for: Muscle synergy stability and human balance maintenance
Source: J Neuroeng Rehabil. 2014 Aug 30;11:129. doi: 10.1186/1743-0003-11-129 (PMC4161771; doi:10.1186/1743-0003-11-129)

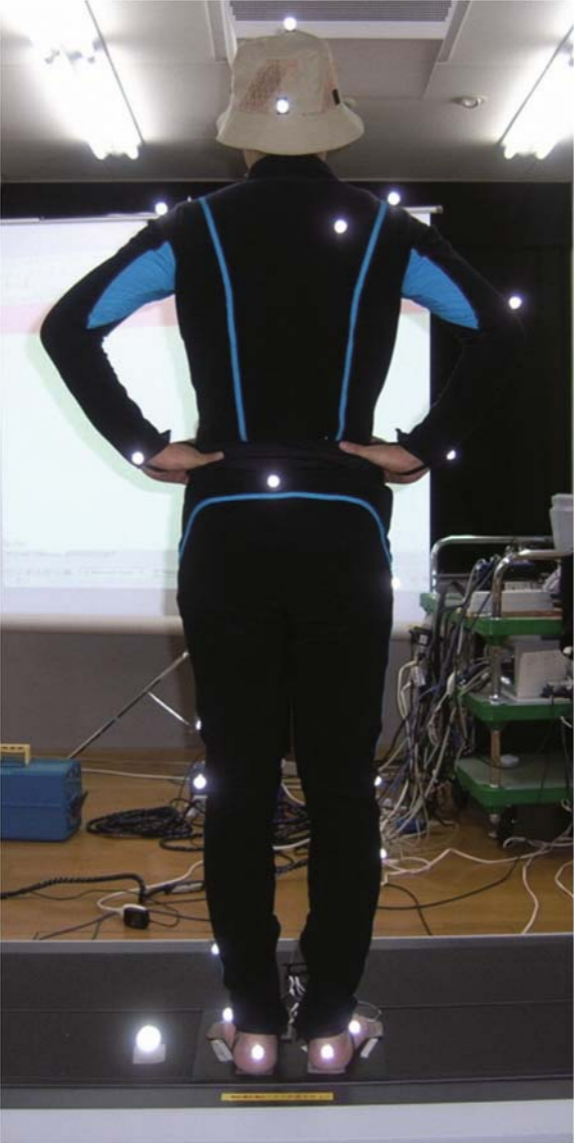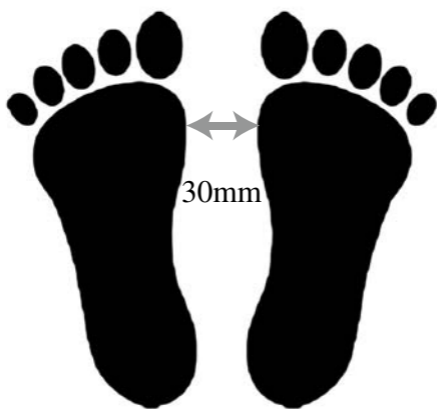

Supplement: Supplementary file 2 — Authors’ original file for figure 1 [file 12984_2013_651_MOESM2_ESM.pdf]

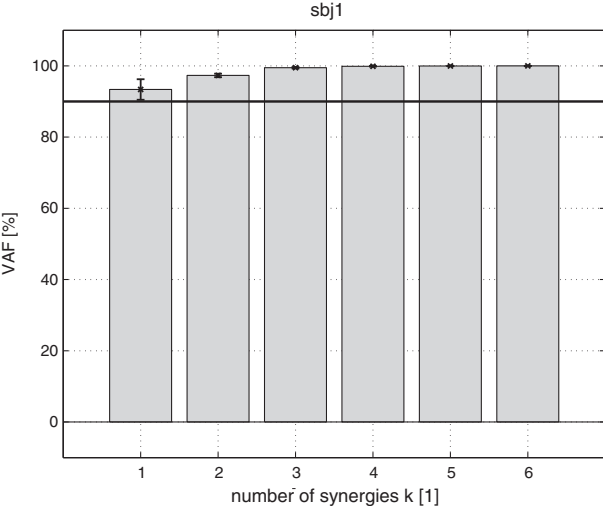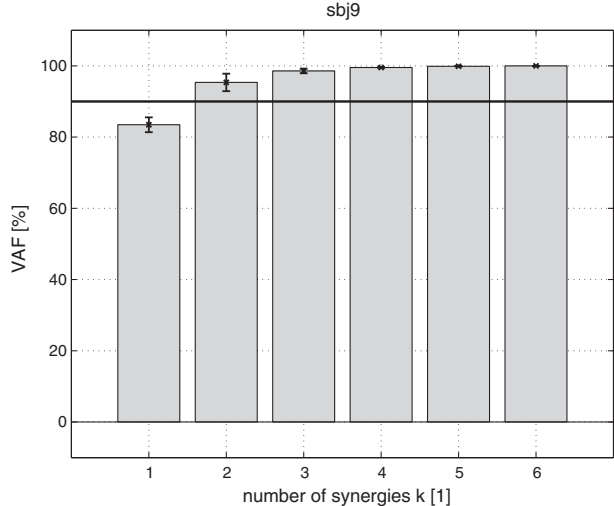

Supplement: Supplementary file 3 — Authors’ original file for figure 2 [file 12984_2013_651_MOESM3_ESM.pdf]

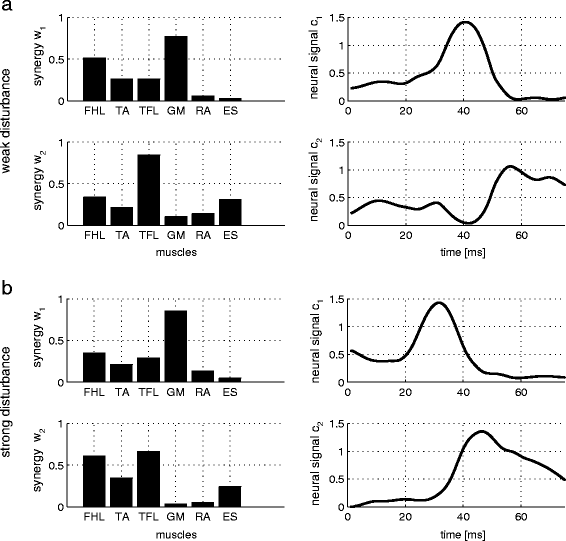

Supplement: Supplementary file 4 — Authors’ original file for figure 3 [file 12984_2013_651_MOESM4_ESM.gif]

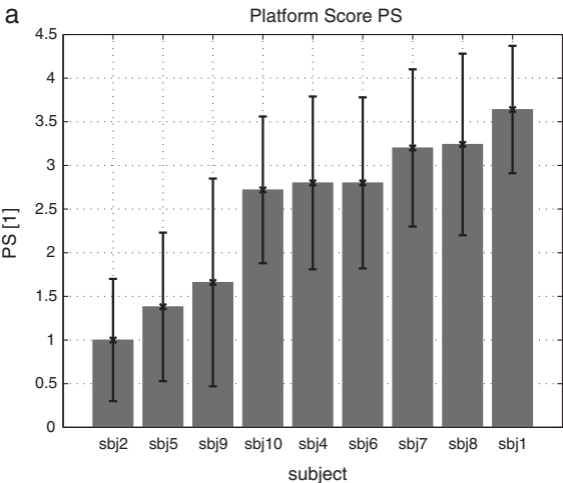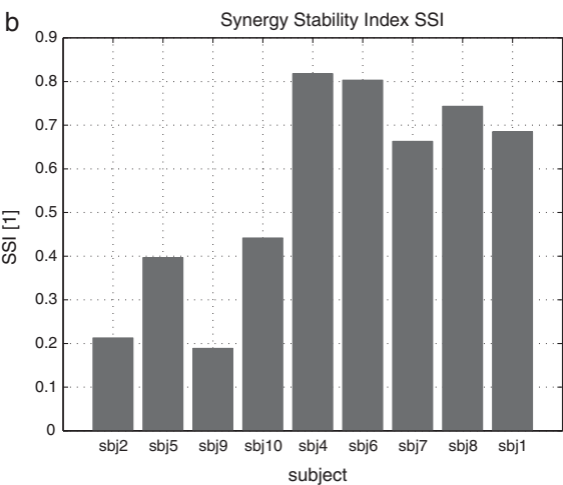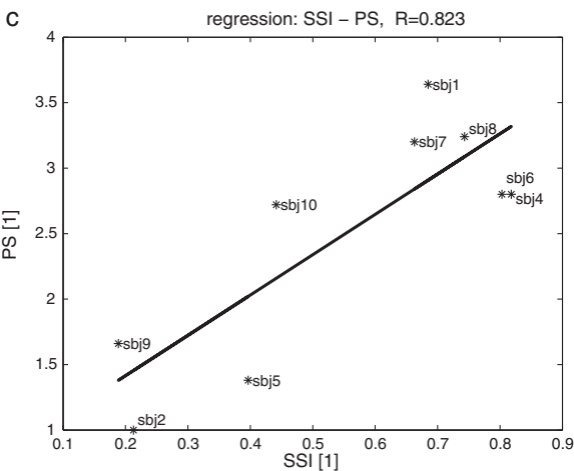

Supplement: Supplementary file 6 — Authors’ original file for figure 5 [file 12984_2013_651_MOESM6_ESM.pdf]

Platform Score (PS) - age,  $R=-0.688$

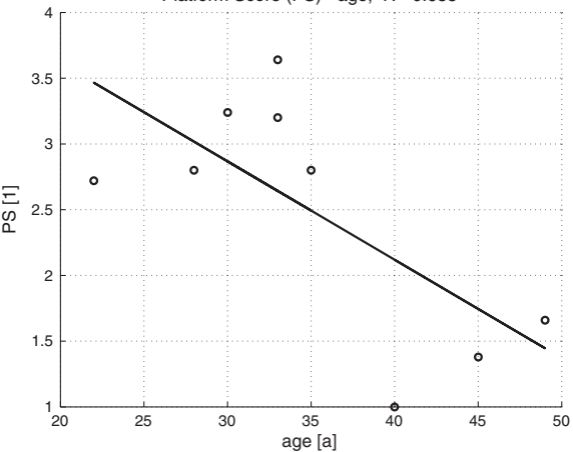

Platform Score (PS) - height,  $R=0.228$

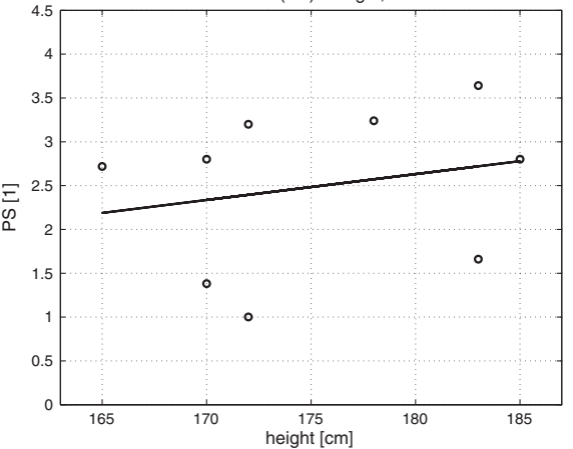

Platform Score (PS) - weight,  $R=0.167$

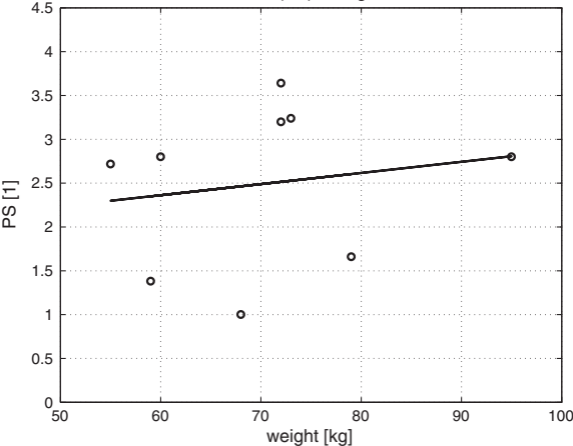

Supplement: Supplementary file 7 — Authors’ original file for figure 6 [file 12984_2013_651_MOESM7_ESM.pdf]
